# Satellite Laser Ranging for Retrieval of the Local Values of the Love *h*_2_ and Shida *l*_2_ Numbers for the Australian ILRS Stations

**DOI:** 10.3390/s20236851

**Published:** 2020-11-30

**Authors:** Marcin Jagoda, Miłosława Rutkowska, Paweł Lejba, Jacek Katzer, Romuald Obuchovski, Dominykas Šlikas

**Affiliations:** 1 Faculty of Civil Engineering, Environmental and Geodetic Sciences, Koszalin University of Technology, Śniadeckich 2, 75-453 Koszalin, Poland; miloslawa.rutkowska@tu.koszalin.pl; 2 Space Research Centre, Polish Academy of Sciences, Bartycka 18A, 00-716 Warsaw, Poland; plejba@cbk.poznan.pl; 3 Faculty of Geoengineering, University of Warmia and Mazury in Olsztyn, Prawocheńskiego 15, 10-720 Olsztyn, Poland; jacek.katzer@uwm.edu.pl; 4 Department of Geodesy and Cadastre, Vilnius Gediminas Technical University, Saulėtekio 11, LT-10233 Vilnius, Lithuania; romuald.obuchovski@vgtu.lt (R.O.); dominykas.slikas@vgtu.lt (D.Š.)

**Keywords:** Love/Shida numbers, satellite laser ranging (SLR), Yarragadee station, Mount Stromlo station, LAGEOS, STELLA, STARLETTE satellites, SLR stations coordinates, ITRF2014

## Abstract

This paper deals with the analysis of local Love and Shida numbers (parameters *h*_2_ and *l*_2_) values of the Australian Yarragadee and Mount Stromlo satellite laser ranging (SLR) stations. The research was conducted based on data from the Medium Earth Orbit (MEO) satellites, LAGEOS-1 and LAGEOS-2, and Low Earth Orbit (LEO) satellites, STELLA and STARLETTE. Data from a 60-month time interval, from 01.01.2014 to 01.01.2019, was used. In the first research stage, the Love and Shida numbers values were determined separately from observations of each satellite; the obtained values of *h*_2_, *l*_2_ exhibit a high degree of compliance, and the differences do not exceed formal error values. At this stage, we found that it was not possible to determine *l*_2_ from the data of STELLA and STARLETTE. In the second research stage, we combined the satellite observations of MEO (LAGEOS-1+LAGEOS-2) and LEO (STELLA+STARLETTE) and redefined the *h*_2_, *l*_2_ parameters. The final values were adopted, and further analyses were made based on the values obtained from the combined observations. For the Yarragadee station, local *h*_2_ = 0.5756 ± 0.0005 and *l*_2_ = 0.0751 ± 0.0002 values were obtained from LAGEOS-1 + LAGEOS-2 and *h*_2_ = 0.5742 ± 0.0015 were obtained from STELLA+STARLETTE data. For the Mount Stromlo station, we obtained the local *h*_2_ = 0.5601 ± 0.0006 and *l*_2_ = 0.0637 ± 0.0003 values from LAGEOS-1+LAGEOS-2 and *h*_2_ = 0.5618 ± 0.0017 from STELLA + STARLETTE. We found discrepancies between the local parameters determined for the Yarragadee and Mount Stromlo stations and the commonly used values of the *h*_2_, *l*_2_ parameters averaged for the whole Earth (so-called global nominal parameters). The sequential equalization method was used for the analysis, which allowed to determine the minimum time interval necessary to obtain stable *h*_2_, *l*_2_ values. It turned out to be about 50 months. Additionally, we investigated the impact of the use of local values of the Love/Shida numbers on the determination of the Yarragadee and Mount Stromlo station coordinates. We proposed to determine the stations (*X*, *Y*, *Z*) coordinates in International Terrestrial Reference Frame 2014 (ITRF2014) in two computational versions: using global nominal *h*_2_, *l*_2_ values and local *h*_2_, *l*_2_ values calculated during this research. We found that the use of the local values of the *h*_2_, *l*_2_ parameters in the process of determining the stations coordinates influences the result.

## 1. Introduction

There are different kinds of external forces acting on the Earth which cause its gradual changes; for this reason, our planet needs constant monitoring. One of these forces is tidal forces, which are reflected, among other things, in the displacement of Earth’s masses and, consequently, in changes in the position of points on the Earth’s surface. To describe the flexible reaction of the Earth to tidal stresses, the concept of so-called Love (*h*, *k*) and Shida (*l*) numbers were introduced. These are tidal parameters whose detailed description was presented in the fundamental works of A.E.H. Love “Some problems of geodynamics” [1] and T. Shida and M. Matsoyama “Note of Hecker’s observations” [2].

The Earth’s dynamics is currently studied using satellite measurement techniques, including the satellite laser ranging (SLR) technique [3]. In our earlier research programme, e.g., [4,5,6], we have successfully demonstrated that the SLR technique makes it possible to determine tidal parameters with very high accuracy, it was also indicated in [7]. Other satellite measuring techniques can also be used for such purposes, e.g., the VLBI technique [8,9]; satellite altimetry [10,11]. All these publications are focused on determining the global values of tidal parameters averaged over the whole Earth.

Due to the heterogeneous structure of our planet, it is reasonable that the reaction to tidal stresses is not the same for the whole Earth. With this in mind, we have launched a research programme to analyze the local tidal parameters. The research carried out so far focused on the Baltic Sea region [12,13], where local tidal parameters for the SLR stations from Poland and Latvia were analyzed based on data from the LAGEOS-1 and LAGEOS-2 satellites. In this study, we made an attempt to determine and analyze local values of tidal parameters for two Australian SLR stations: Yarragadee (no. 70900513, approx. 29° S, 115° E) and Mount Stromlo (no. 78259001, approx. 35° S, 149° E). In addition, we assessed the impact of their use on the determination of the coordinates of these stations. These tasks constitute the main research objective of this work. Estimation of the minimum time interval ensuring the stability of the determination and the assessment of the possibility of determining local tidal parameters from the data of the LEO satellites STELLA and STARLETTE constitute the intermediary purpose of this study.

**Figure 1:**
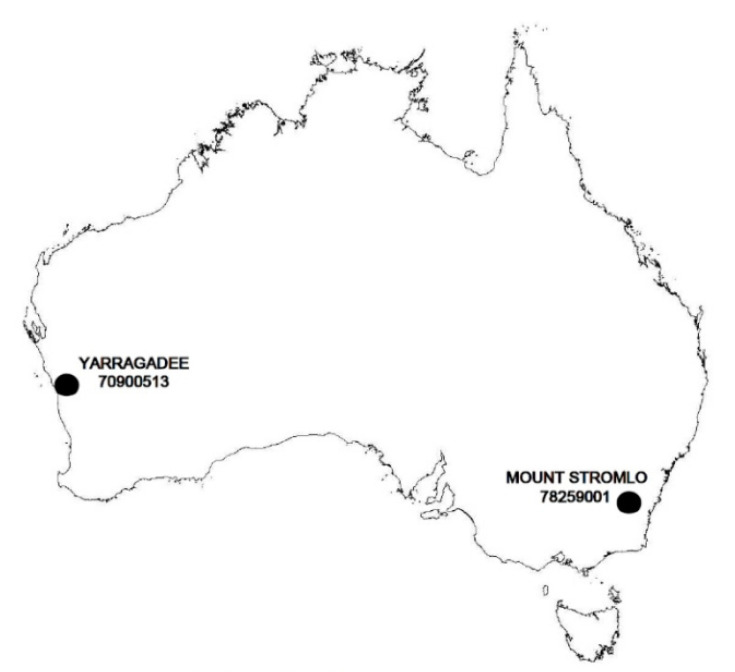
Location of the Australian satellite laser ranging (SLR) stations.

The data provided by the Australian Yarragadee and Mount Stromlo stations are extremely important for geodynamic research. The global SLR network consists of 38 stations, of which only eight are located in the Southern Hemisphere; two of them on the Australian continent. Their location is shown in Figure 1. 

The Yarragadee station is located in Western Australia, near the city of Dongara. The Mount Stromlo satellite laser ranging observatory is located in the south-eastern part of the continent, near the city of Geraldton. These stations are part of the Western Pacific Laser Tracking Network and contribute to the International Laser Ranging Service (ILRS). These are some of the best stations in terms of accuracy and number of observations. The data they collect plays a very important role, in synergy with other geodetic techniques, in defining International Terrestrial Reference Frame (ITRF) and determining Earth Orientation Parameters (EOP).

The basis of the satellite laser ranging technique (SLR) is the measurement of two-way time of light pulses flight between a station and a satellite fitted with retroreflectors. The distance measured to the satellite must be adjusted to accommodate the effects of a speed of light decrease and the difference between the straight and curved paths of ray. Furthermore, it must take into consideration the distance from the retroreflector to the satellite mass center, influence of the satellite motion, the Earth rotation and relativistic effects [14]. In its simplified form, the equation of laser observation is as follows [14]:

(1) $$\rho =\frac{C\Delta t}{2}$$

where ρ is the distance between a station and a satellite, Δt is the two way time interval of light pulses flight between a station and a satellite, and *C* is the speed of light.

Typical, geodetic SLR satellites are sphere-shaped, covered with retroreflectors and can be divided into two main groups: Medium Earth Orbit (MEO) satellites; e.g., LAGEOS-1 (Perige = 5860 km), LAGEOS-2 (Perige = 5620 km); and Low Earth Orbit (LEO) satellites, e.g., STELLA (Perige = 804 km) and STARLETTE (H = 812 km). The data from these satellites is widely used in geodynamic research; e.g., to determine stations coordinates [15,16,17], to study the gravitational field of the Earth [18], to determine Earth Orientation Parameters [19,20,21,22], or to study the tidal phenomenon [23,24]. In this work, we used the data of the LAGEOS-1, LAGEOS-2, STELLA, and STARLETTE satellites to determine the local values of the tidal parameters and coordinates of the Australian SLR Yarragadee and Mount Stromlo stations. A detailed description of the SLR technique can be found in the works [25,26], while a wide range of applications of laser satellites in geodynamic research has been presented in [27,28,29]. 

The gravitational impact of the Moon, the Sun, and the Solar System planets on the Earth’s surface results in the creation of earth and ocean tides. The tidal forces cause the displacement of earth and ocean masses. A detailed description of the tide phenomenon and its mathematical basis can be found in fundamental work “The tides of the planet Earth” by P. Melchior [30]. These changes in the distribution of the Earth’s masses related to tides are expressed by movements of observation stations, as described by Equation (2) given in [31]:

(2) $$\begin{array}{l} \Delta X=\sum_{j=2}^{3}\left[\frac{GM_{j}}{GM_{E}}\frac{a_{e}^{4}}{d_{j}^{3}}\right]\left\{\left[3l_{2}\left({\hat{R}}_{j}{\hat{r}}_{sta}\right)\right]\bar{X}{}_{j}+\left[3\left(\frac{h_{2}}{2}-l_{2}\right){\left({\hat{R}}_{j}{\hat{r}}_{sta}\right)}^{2}-\frac{h_{2}}{2}\right]{\bar{X}}_{sta}\right\}\\ \Delta Y=\sum_{j=2}^{3}\left[\frac{GM_{j}}{GM_{E}}\frac{a_{e}^{4}}{d_{j}^{3}}\right]\left\{\left[3l_{2}\left({\hat{R}}_{j}{\hat{r}}_{sta}\right)\right]\bar{Y}{}_{j}+\left[3\left(\frac{h_{2}}{2}-l_{2}\right){\left({\hat{R}}_{j}{\hat{r}}_{sta}\right)}^{2}-\frac{h_{2}}{2}\right]{\bar{Y}}_{sta}\right\}\\ \Delta Z=\sum_{j=2}^{3}\left[\frac{GM_{j}}{GM_{E}}\frac{a_{e}^{4}}{d_{j}^{3}}\right]\left\{\left[3l_{2}\left({\hat{R}}_{j}{\hat{r}}_{sta}\right)\right]\bar{Z}{}_{j}+\left[3\left(\frac{h_{2}}{2}-l_{2}\right){\left({\hat{R}}_{j}{\hat{r}}_{sta}\right)}^{2}-\frac{h_{2}}{2}\right]{\bar{Z}}_{sta}\right\} \end{array}$$

where

$GM_{j}$—gravitational parameter for the Moon (*j* = 2) or the Sun (*j* = 3),
$GM_{E}$—gravitational parameter for the Earth,
*a_e_*—equatorial radius,
*d_j_*—distance to the Moon (*j* = 2) or Sun (*j* = 3),
${\hat{R}}_{j}$—the unit vector from the geocenter to the Moon (*j* = 2) or Sun (*j* = 3),
${\hat{r}}_{sta}$—the unit vector from the geocenter to the station,
$\left({\bar{X}}_{j},{\bar{Y}}_{j},{\bar{Z}}_{j}\right)$—the Cartesian components of the unit vector ${\hat{R}}_{j}$,
$\left({\bar{X}}_{sta},{\bar{Y}}_{sta},{\bar{Z}}_{sta}\right)$—the Cartesian components of the unit vector $\hat{r}$,
*h*_2_, *l*_2_—second degree of Love and Shida numbers.

In Equation (2), there are the tidal parameters *h*_2_ and *l*_2_ (Love and Shida numbers for the second-degree tides). The former refers to the radial tidal displacement of the station, the latter to the horizontal displacement, it is described in [32]. The tidal parameters are a measure of the flexible Earth’s response to stresses created by tidal forces. If we assume that the Earth is a rigid body, then no elastic deformation takes place, and *h*, *l* are both 0. If we assume another extreme case in which the Earth is not just elastic but rather a liquid body, then the Love and Shida numbers are both equal to 1. Thus, for a rigid Earth *h* = 0, *l* = 0, for a liquid Earth *h* = 1, *l* = 1 and for an elastic Earth they take intermediate values: 0 < *h* < 1, 0 < *l* < 1. According to International Earth Rotation and Reference Systems Service (IERS) Conventions (IERS Technical Note No. 36) [33], the Earth’s global (so called nominal) averaged values of the Love and Shida numbers for the second degree tides are *h*_2_ = 0.6078, *l*_2_ = 0.0847.

## 2. Materials and Methods

To determine the local tidal parameters of the Australian Yarragadee and Mount Stromlo stations, we used observation data in the form of normal points of these stations collected for the LAGEOS-1, LAGEOS-2, STELLA, and STARLETTE satellites for the 5-year interval from 01.01.2014 to 01.01.2019. For the Yarragadee station, these were respectively: 57,299 LAGEOS-1 normal points, 58,133 LAGEOS-2 normal points, 38,031 STELLA normal points, 90,953 STARLETTE normal points; for the Mount Stromlo station: 25,249 LAGEOS-1 normal points, 25,962 LAGEOS-2 normal points, 21,654 STELLA normal points, 50,172 STARLETTE normal points. The method of creating normal points from SLR measurements is described in [34]. The data from the analyzed period were used to create 7-day orbital arcs. In total, 260 orbital arcs were obtained for each of the satellites. Satellite orbits were determined using the Cowell Numerical Integration method as described in detail in [31], using standard procedures, force models and constants recommended by the International Earth Rotation and Reference Systems Service (IERS) [33] and International Laser Ranging Service (ILRS) [35]. RMS values of the post-fit residuals, calculated from formula (3), were used as the satellites orbits accuracy determination [5]:

(3) $$\mathrm{RMS}\;\mathrm{of}\;\mathrm{the}\;\mathrm{post}\text{-}\mathrm{fit}\;\mathrm{residuals}\;=\sqrt{\frac{\sum_{i=1}^{n}{\left(O_{i}-C_{i}\right)}^{2}}{n-1}}$$

where *i* denotes successive number of normal points, (*O_i_* − *C_i_*) is the SLR observation minus the computed distance from the station to the satellite. The following values were obtained: RMS of the post-fit residuals: RMS(LAGEOS-1) = 1.02 cm, RMS(LAGEOS-2) = 1.01 cm, RMS(STELLA) = 1.98 cm, RMS(STARLETTE) = 1.87 cm.

To determine the local tidal parameters *h*_2_, *l*_2_ values of the Yarragadee and Mount Stromlo stations and their coordinates, an observation Equation (4) was formulated and solved using the Bayesian least square method, a detailed description of this procedure is given in [31]. The local tidal parameters and coordinates were determined independently for both of the analyzed stations.

(4) $$(O_{i}-C_{i})=-\;\;\left\{\sum_{j=1}^{n}\frac{\partial C_{i}^{}}{\partial \varepsilon_{j}}d\varepsilon_{j}+\frac{\partial C_{i}^{}}{\partial h_{2}}dh_{2}+\frac{\partial C_{i}^{}}{\partial l_{2}}dl_{2}\right\}+dO_{i}$$

where

j—number of adjusted parameters (satellite position and velocity, empirical accelerations, and the station position),
$d\varepsilon_{j}$—corrections to the *j*-th parameter,
$dh_{2}$, $dl_{2}$—corrections for Love number *h*_2_ and for Shida number *l*_2_,
$dO_{i}$—error of observation associated with the *i*-th measurement.

Given in Equation (4) the $\frac{\partial C_{i}^{}}{\partial h_{2}},\frac{\partial C_{i}^{}}{\partial l_{2}}$ quantities are calculated by differentiating Equation (2) and are expressed as follows [31]:

(5) $$\frac{\partial C_{i}^{}}{\partial h_{2}}=\frac{\partial C_{i}^{}}{\partial X_{sta}}\frac{\partial X_{sta}}{\partial h_{2}}+\frac{\partial C_{i}^{}}{\partial Y_{sta}}\frac{\partial Y_{sta}}{\partial h_{2}}+\frac{\partial C_{i}^{}}{\partial Z_{sta}}\frac{\partial Z_{sta}}{\partial h_{2}}$$

(6) $$\frac{\partial C_{i}^{}}{\partial l_{2}}=\frac{\partial C_{i}^{}}{\partial X_{sta}}\frac{\partial X_{sta}}{\partial l_{2}}+\frac{\partial C_{i}^{}}{\partial Y_{sta}}\frac{\partial Y_{sta}}{\partial l_{2}}+\frac{\partial C_{i}^{}}{\partial Z_{sta}}\frac{\partial Z_{sta}}{\partial l_{2}}$$

where

(7) $$\begin{array}{l} \frac{\partial X_{sta}}{\partial h_{2}}=\sum_{j=2}^{3}\frac{GM_{j}}{GM_{E}}\frac{a_{e}^{4}}{d_{j}^{3}}\left[\frac{3}{2a_{e}}{\left({\hat{R}}_{j}{\hat{r}}_{st}\right)}^{2}-\frac{1}{3}\right]X_{sta},\\ \frac{\partial Y_{sta}}{\partial h_{2}}=\sum_{j=2}^{3}\frac{GM_{j}}{GM_{E}}\frac{a_{e}^{4}}{d_{j}^{3}}\left[\frac{3}{2a_{e}}{\left({\hat{R}}_{j}{\hat{r}}_{st}\right)}^{2}-\frac{1}{3}\right]Y_{sta},\\ \frac{\partial Z_{sta}}{\partial h_{2}}=\sum_{j=2}^{3}\frac{GM_{j}}{GM_{E}}\frac{a_{e}^{4}}{d_{j}^{3}}\left[\frac{3}{2a_{e}}{\left({\hat{R}}_{j}{\hat{r}}_{st}\right)}^{2}-\frac{1}{3}\right]Z_{sta}, \end{array}$$

(8) $$\begin{array}{c} \frac{\partial X_{sta}}{\partial l_{2}}=3\sum_{j=2}^{3}\frac{GM_{j}}{GM_{E}}\frac{a_{e}^{4}}{d_{j}^{3}}\left[3({\hat{r}}_{st}{\hat{R}}_{j}){\bar{X}}_{j}-\frac{1}{a_{e}}{\left({\hat{R}}_{j}{\hat{r}}_{st}\right)}^{2}X_{sta}\right],\\ \frac{\partial Y_{sta}}{\partial l_{2}}=3\sum_{j=2}^{3}\frac{GM_{j}}{GM_{E}}\frac{a_{e}^{4}}{d_{j}^{3}}\left[3({\hat{r}}_{st}{\hat{R}}_{j}){\bar{Y}}_{j}-\frac{1}{a_{e}}{\left({\hat{R}}_{j}{\hat{r}}_{st}\right)}^{2}Y_{sta}\right],\\ \frac{\partial Z_{sta}}{\partial l_{2}}=3\sum_{j=2}^{3}\frac{GM_{j}}{GM_{E}}\frac{a_{e}^{4}}{d_{j}^{3}}\left[3({\hat{r}}_{st}{\hat{R}}_{j}){\bar{Z}}_{j}-\frac{1}{a_{e}}{\left({\hat{R}}_{j}{\hat{r}}_{st}\right)}^{2}Z_{sta}\right]. \end{array}$$

The sequential method was used to determine local tidal parameters. In the first step, the *h*_2_ and *l*_2_ parameters were determined separately from each orbital arc (arc1, arc2, arc3, …, arc260). The following steps consisted in adding subsequent arcs to the calculations, one after another, following the scheme: arc1 + arc2, arc1 + arc2 + arc3, …, arc1 + arc2 + … + arc260. In each subsequent step, *h*_2_ and *l*_2_ parameters were re-computed. The values given in IERS Technical Note No. 36 [33] were taken as priori values (*h*_2_ = 0.6078 and *l*_2_ = 0.0847). In the first calculation stage, the local tidal parameters were determined separately from LAGEOS-1, LAGEOS-2, STELLA, and STARLETTE data, then data from LAGEOS-1 and LAGEOS-2 and STELLA and STARLETTE were pooled (LAGEOS-1+LAGEOS-2 and STELLA+STARLETTE) and re-computed to increase the accuracy and stability of the solutions. The final values were adopted and further analyses were made based on the values obtained from the combined observations of 260 orbital arcs.

Additionally, the coordinates of the Yarragadee and Mount Stromlo stations were determined in course of the analysis. These coordinates were calculated from the Equation (4). The determination method of the stations’ coordinates from the SLR data was set out in detail in [14,36]. The coordinates of the Yarragadee and Mount Stromlo stations were determined from the LAGEOS-1 + LAGEOS-2 data with a presumptive assumption of the stations coordinates in the ITRF2014 reference frame [37]. The adjustment was performed in two calculation versions. As regards the first one, Yarragadee and Mount Stromlo stations coordinates were calculated using of the global nominal values (recommended in the IERS Conventions [33]) of tidal parameters. In the second one, Yarragadee and Mount Stromlo stations coordinates were estimated using the local values of tidal parameters calculated in this present paper. The impact of the application of different values of tidal parameters on the determination of these stations coordinates was then investigated.

The GEODYN II NASA GSFC software [31] was used for all the calculations related to the determination of satellite orbits, local tidal parameters and coordinates of Yarragadee and Mount Stromlo SLR stations.

## 3. Results and Discussion

**Figure 2:**
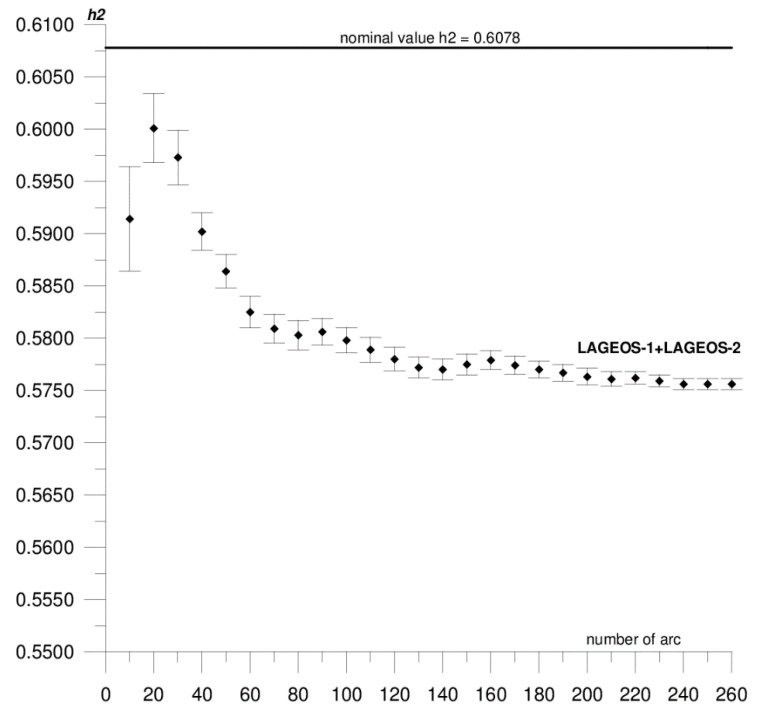
Sequential solution for the Yarragadee local *h*_2_ parameter based on LAGEOS-1+LAGEOS-2 data.

**Figure 3:**
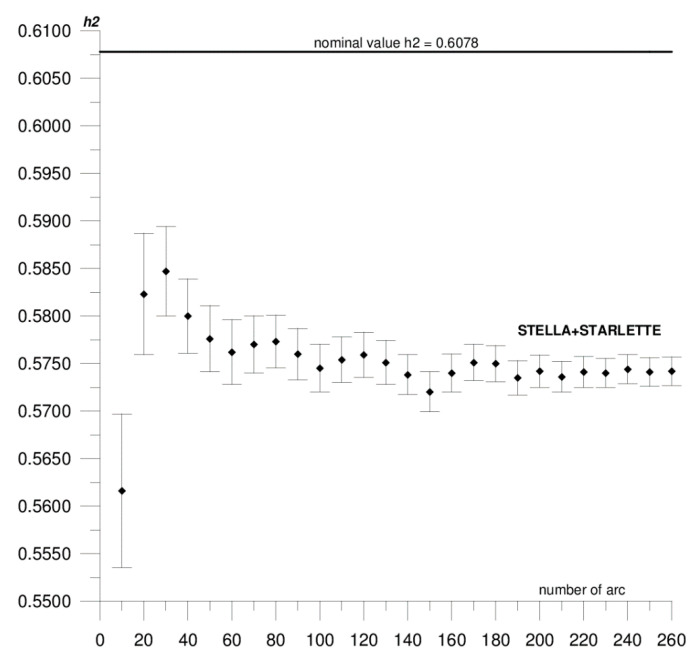
Sequential solution for the Yarragadee local *h*_2_ parameter based on STELLA+STARLETTE data.

**Figure 4:**
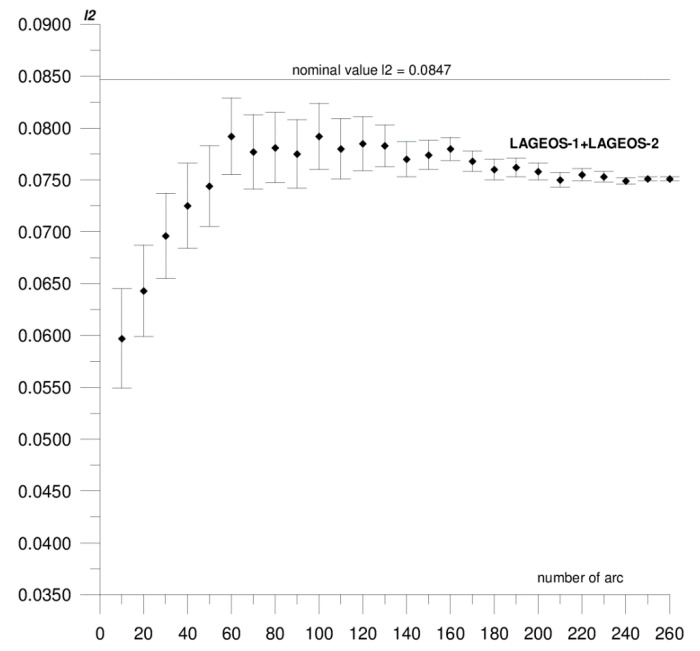
Sequential solution for the Yarragadee local *l*_2_ parameter based on LAGEOS-1+LAGEOS-2 data.

**Figure 5:**
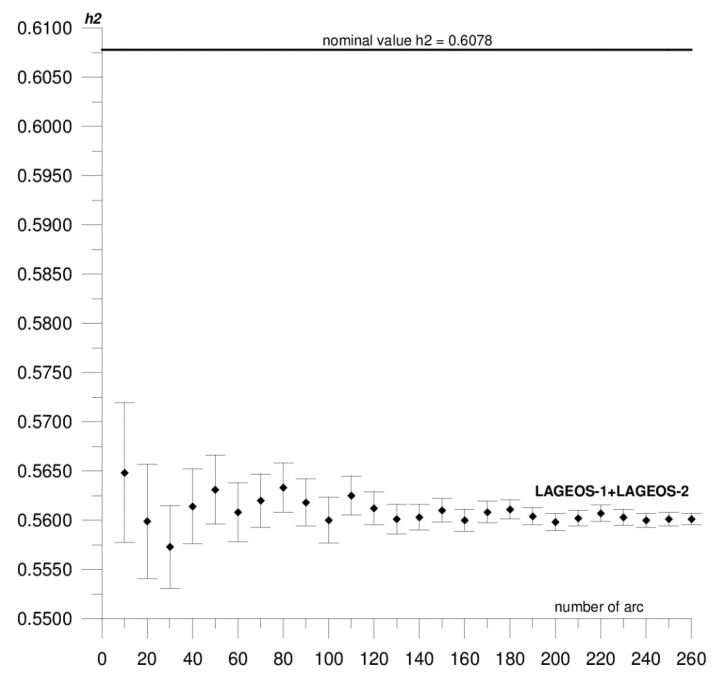
Sequential solution for the Mount Stromlo local *h*_2_ parameter based on LAGEOS-1+LAGEOS-2 data.

**Figure 6:**
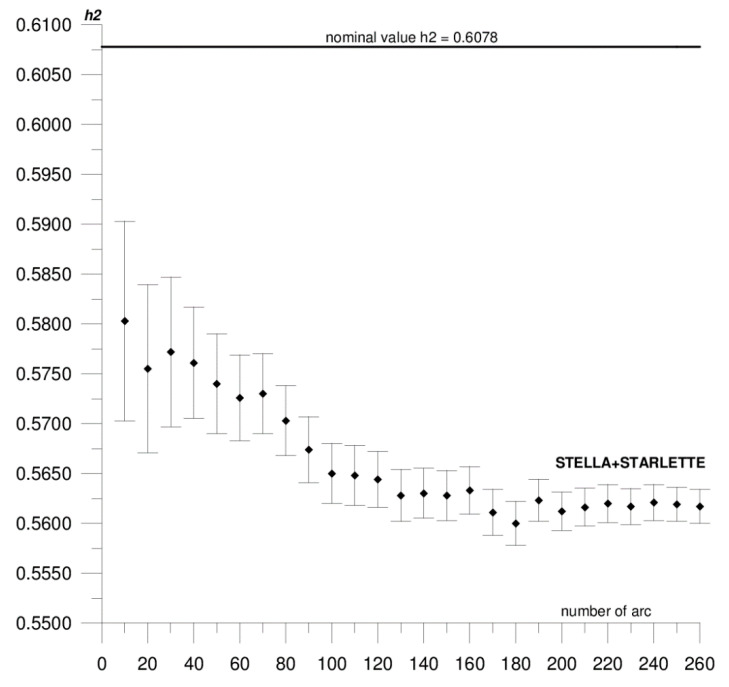
Sequential solution for the Mount Stromlo local *h*_2_ parameter based on STELLA+STARLETTE data.

**Figure 7:**
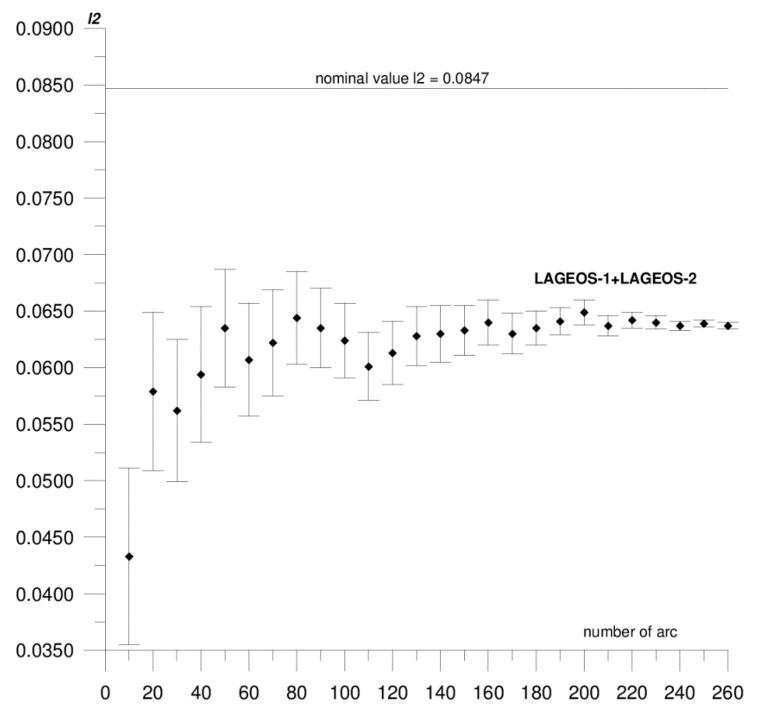
Sequential solution for the Mount Stromlo local *l*_2_ parameter based on LAGEOS-1+LAGEOS-2 data.

In this paper, we present the results of the determination of the local values of tidal parameters *h*_2_, *l*_2_ for the Australian SLR stations Yarragadee and Mount Stromlo, and their coordinates in the ITRF2014 reference frame [37]. The first stage of the research included determining the local tidal parameters separately from the data of each of the satellites: LAGEOS-1, LAGEOS-2, STELLA, and STARLETTE. The obtained *h*_2_, *l*_2_ values show a high degree of consistency, and the differences do not exceed formal error values (please refer to Table 1). At this stage, we found that it was not possible to determine *l*_2_ from STELLA and STARLETTE data. Then, to increase the accuracy and stability of the solutions, we pooled the data of the individual satellite groups, LAGEOS-1+LAGEOS-2 and STELLA+STARLETTE, and re-computed the local tidal parameters. We did not determine the *l*_2_ parameter from STELLA+STARLETTE data. The values obtained in this way were assumed final and subjected to further analysis. The final estimated values of the local tidal parameters for the Yarragadee and Mount Stromlo stations are given in Table 1, whereas the results of the sequential determination method are shown in Figure 2, Figure 3, Figure 4, Figure 5, Figure 6 and Figure 7. For clarity and readability of the figures, we present results orbital arcs combined in groups of ten (arcs 1–10, 1–20, 1–30, …, 1–260).

In the first step of the sequential method, the *h*_2_, *l*_2_ parameters were determined from two orbital arcs. The computed values significantly deviate from the final ones. Adding arcs in weekly cycles (up to 260) allows the observation of a slowly emerging stability approaching the final *h*_2_, *l*_2_ values determined from the 260 arcs. The values of formal errors of the determined parameters also asymptotically approach their final values. The process of achieving stability varies across parameters and stations. For the Yarragadee station, for the *h*_2_ parameter, the designation stability (understood as the repeatability of the results obtained for subsequently added arcs down to the level of formal error) for LAGEOS-1+LAGEOS-2 data (Figure 2) emerges at about 200 arcs. The situation is similar for the determination from STELLA+STARLETTE data (Figure 3). The *l*_2_ parameter (Figure 4) exhibits a lower degree of determination stability, achieved after about 230 arcs. In turn, for the Mount Stromlo station, the stability of the *h*_2_ parameter determination was achieved for about 190 LAGEOS-1+LAGEOS-2 arcs (Figure 5) and 200 STELLA+STARLETTE arcs (Figure 6). The determination stability of the *l*_2_ parameter for the Mount Stromlo station is similar to that of Yarragadee station, and was achieved after about 230 arcs (Figure 7). It proves that the number of arcs needed to determine local tidal parameters of these stations is about 200, which corresponds to about a 50-month interval (seven-day orbital arcs). For next added arcs, the estimated parameters values vary less than the formal error value.

Figure 2, Figure 3 and Figure 4 show the results of the sequential solution for the Yarragadee station local tidal parameters. The values of the *h*_2_ and *l*_2_ numbers for this station, determined from LAGEOS-1+LAGEOS-2 data are 0.5756 ± 0.0005 and 0.0751±0.0002, respectively, and differ from the global values *h*_2_ and *l*_2_ given in IERS Technical Note No. 36 [33] by 0.0322 (5%) and 0.0096 (11%), respectively. A similar value of the *h*_2_ parameter was obtained from the data of the STELLA+STARLETTE satellites: *h*_2_ = 0.5742 ± 0.0015 (the difference with respect to the global value is 0.0336, i.e., about 6%). The *l*_2_ parameter was not determined due to an unacceptable value and large error obtained when independently determining from the STELLA and SRTARLETTE data (see Table 1). Jagoda and Rutkowska [5], where global values of tidal parameters determined from LEO satellites data were analyzed from January 2005 to July 2007, present similar findings. The values of horizontal displacement of Earth masses in effect of tidal forces which are described by Shida *l*_2_ number are significantly lower and harder to be measured than radial displacements which are expressed by Love *h*_2_ number. This can potentially affect a determination of *l*_2_ parameter from the LEO satellites data.

In general, the results of determining *h*_2_ for the Yarragadee station from STELLA+STARLETTE data are very similar to those from LAGEOS-1+LAGEOS-2, with the difference being 0.0014, i.e., in the range of formal error. 

In turn, the formal error in *h*_2_ designation is three times greater for STELLA+STARLETTE, which is due to the impaired orbit designation of these satellites. The LEO satellites STELLA and STARLETTE move in the lower, dense layers of the atmosphere (at an altitude of about 800 km), and therefore their orbits are determined with greater errors than those of LAGEOS satellites. For the STELLA and STARLETTE satellites in [38] authors obtained mean RMS values of the post-fit residuals from 1.30 cm to 1.87 cm depending on the Earth gravity field model used. In another paper [39], mean RMS values of the post-fit STELLA/STARLETTE were given from 1.87 cm to 2.90 cm depending on the frequency of estimation of empirical acceleration parameters. In [40] these were 3.11 cm for STELLA and 2.40 cm for STARLETTE. In this paper, the mean RMS values of the post-fit STELLA and STARLETTE were 1.98 cm and 1.87 cm, respectively. In turn, the LAGEOS satellite orbits at an altitude of about 6000 km are determined with an accuracy of about 1 cm, and RMS values of the post-fit of this order were obtained, e.g., in [6,39]. The mean RMS values of the post-fit residuals for LAGEOS-1 and LAGEOS-2 obtained in this analysis are 1.02 cm and 1.01 cm, respectively.

Figure 5, Figure 6 and Figure 7 depict the results of sequential solution for Mount Stromlo station local tidal parameters. The values of the tidal parameters for this station determined from the LAGEOS-1+LAGEOS-2 data are *h*_2_ = 0.5601 ± 0.0006, *l*_2_ = 0.0637 ± 0.0003. The differences with respect to global values are 0.0477 (8%) for *h*_2_ and 0.021 (25%) for *l*_2_. The value of the *h*_2_ parameter for this station determined from the STELLA+STARLETTE data is 0.5618 ± 0.0017, the difference from the nominal value is 0.046, that is about 7%. Similarly, as in the case of the Yarragadee station, the *l*_2_ parameter from combined LEO satellites data was not determined. There is a high degree of conformity between the *h*_2_ values obtained from LAGEOS-1+LAGEOS-2 and STELLA+STARLETTE data, with the difference being 0.0017 and not exceeding the formal error level. Similar to the Yarragadee station, the formal error of the *h*_2_ parameter is higher for the LEO satellites; about three times in this case.

The comparison of Love/Shida numbers for the Yarragadee and Mount Stromlo stations shows that they differ by 0.0155 ± 0.0005 (LAGEOS-1+LAGEOS-2 data) and 0.0124 ± 0.0015 (STELLA+STARLETTE data) for the *h*_2_ number and 0.0114 ± 0.0002 (LAGEOS-1+LAGEOS-2 data) for the *l*_2_ number. These differences exceed the formal error level. So far, no similar studies have been carried out for SLR stations from Australia, so it is impossible to relate the results obtained to the work of other researchers. However, data for two European SLR stations from the Baltic Sea region are available: Borowiec (no. 78113802) and Riga (no. 18844401). Jagoda and Rutkowska [12] were found that the local tidal parameters for the Borowiec station determined from the LAGEOS satellites data in the 01.01.2009–01.01.2019 interval are *h*_2_ = 0.7308 ± 0.0008 and *l*_2_ = 0.1226 ± 0.0003. In another paper Jagoda and Rutkowska [13], were obtained *h*_2_ = 0.6891 ± 0.0009 and *l*_2_ = 0.1043 ± 0.0004 for the Riga station from the LAGEOS satellites data in the 01.01.2004–01.01.2019 interval. Significant differences can be found when comparing the results obtained in this work to the results for the European stations Borowiec and Riga. These are the largest for Mount Stromlo and Borowiec stations: about 23% for the *h*_2_ parameter and about 48% for the *l*_2_ parameter.

The differences between the global and local tidal parameters may be influenced by the geological structure and physical factors of the observation site, in this case Australia. The Australian continent is located in the eastern part of the Indo–Australian lithosphere plateau. The greater part of Australia is occupied by the Precambrian Craton called the Australian Craton, which is adjacent to the structure of the Flinders Ranges and the Barrier Ranges, and the structure of the Great Dividing Range [41]. The Yarragadee station is located within the Australian Craton on the so-called Perth Basin. The Perth Basin is filled mainly with continental Permian sediments which lie directly on crystalline rocks. The rocks of the Perth Basin sedimentary cover are mainly sandy-loam and marine sediments of Triassic and Cretaceous periods [42,43]. The eastern part of Australia where the Mount Stromlo station is located, is occupied by the of the Great Dividing Range. The Palaeozoic structures of the Great Dividing Range were created as a result of subduction processes on the border between Panthalassa and the Gondwana Craton, on the fringe of which the Australian continent was situated [42]. The mountain range created as a result of these processes is characterised by a varied structure and geological history. In the western part, the structures formed in the Neoproterozoic era region dominate. In the central part, the main phases of the tectonic movements, magmatism, and metamorphism were at work from the early Ordovician to the lowest Devonian. In the eastern part, on the rocks of the older Palaeozoic there are thick sediments of the Devonian and the lower Carboniferous as well as the Permian. The tectonic movements lasted here from the Carboniferous. They were accompanied by lava outflows, the covers of which amount to many thousands of square kilometers of the area. In numerous places, after the fold movements, tectonic subsidences and grabens were formed [42,43].

In addition we have studied the impact of adjusted local tidal parameters *h*_2_, *l*_2_ values on the determination of the Yarragadee and Mount Stromlo SLR stations coordinates in the ITRF2014 reference frame [37]. The test consisted in determining the *X*, *Y*, *Z* coordinates of Yarragadee and Mount Stromlo stations in ITRF2014 in two computational versions. In the first computational version, the coordinates were determined using the nominal global values *h*_2_ = 0.6078, *l*_2_ = 0.0847 [33]. The second version consisted in determining the coordinates using the proposed in this analysis local tidal parameters *h*_2_ =0.5756, *l*_2_ = 0.0751 for the Yarragadee station and *h*_2_ = 0.5601, *l*_2_ = 0.0637 for the Mount Stromlo station. Table 2 presents the test results.

The use of local tidal parameters *h*_2_, *l*_2_ values instead of global nominal *h*_2_, *l*_2_ values affects the result of the coordinate determination. The *Z* coordinate seems to be the most affected one, with the difference between version 1 and 2 being 0.0055 m and −0.0052 m for Mount Stromlo and Yarragadee stations, respectively. The smallest difference was observed for the *X* coordinate: −0.0033 m for Yarragadee and −0.0038 m for Mount Stromlo. The *Y* component differed by 0.0041 m (Yarragadee) and 0.0050 m (Mount Stromlo). In [12], in a similar test performed for the Borowiec station, the same order of differences was obtained (Δ*X* = −0.0035 m, Δ*Y* = 0.0033 m, Δ*Z* = 0.0042 m) as for the Yarragadee and Mount Stromlo stations. However, in [13] describing the Riga station, these discrepancies are larger, namely, Δ*X* = 0.0044 m, Δ*Y* = −0.0047 m, Δ*Z* = 0.0069 m. 

Similar results of determining the coordinates of the Yarragadee and Mount Stromlo stations in ITRF2014 system were obtained in the paper [17], where the authors proposed a kinematic method to estimate the coordinates of SLR stations by using the Global Navigation Satellite System (GNSS) technique onboard a low Earth orbiting (LEO) satellite. They applied SLR and GNSS observations of the GRACE-A satellite from January to December 2012. They found that the GRACE-A satellite, as a connection between the SLR and GNSS techniques, allowed the accurate estimation of SLR stations positions with the high agreement with the ITRF2014 system.

In another paper [22], the author used the STARLETTE, LAGEOS-1 and LAGEOS-2 data over a 14-year period (1993–2007) for determination and analysis of SLR stations coordinates in ITRF2005 system [44]. The author pointed out a good agreement of the estimated coordinates with respect to the values given in ITRF2005. However, in both of these studies the influence of the application of different values of *h*_2_, *l*_2_ parameters on the results of determining the SLR stations coordinates was not investigated.

## 4. Conclusions

Based on the results obtained in the considered case studies, the following conclusions can be drawn:

- There are discrepancies observed between the determined local tidal parameters *h*_2_, *l*_2_ for the Yarragadee and Mount Stromlo stations and the commonly used values of the *h*_2_, *l*_2_ parameters averaged for the whole Earth. This may be influenced by the geological structure and physical factors of the observation site. In order to confirm this, detailed geophysical analyses should be carried out. This goes beyond the scope of this work, suggesting at the same time the need for further studies in this field.
- The use of local tidal parameters values in the process of determining the stations coordinates influences the result.
- Local tidal parameters *h*_2_, *l*_2_ are better determined from the LAGEOS-1 and LAGEOS-2 data than from the STELLA and STARLETTE. However, the results obtained from the LEO satellites indicate that data from these satellites can be used for the determination of local tidal parameters. They can be used for stations with a low number of observations from the LAGEOS satellites.
- It is not possible to determine the *l*_2_ parameter for the Yarragadee and Mount Stromlo stations from STELLA and STARLETTE data. The values of horizontal displacement of Earth masses which are described by the *l*_2_ parameter are significantly lower and harder to be measured than radial displacements which are expressed by the *h*_2_ parameter. This can potentially affect a determination of *l*_2_ parameter from STELLA and STARLETTE data.
- The time interval adopted in the analysis is sufficient to determine the *h*_2_ and *l*_2_ local parameters. The results stabilize after about 200 orbital arcs, which corresponds to about 50 months from the 60-month interval adopted in the analysis.

## Figures and Tables

**Table 1:** Local tidal parameters *h*_2_, *l*_2_ for Yarragadee and Mount Stromlo SLR stations.

SLR Data	Yarragadee (No. 70900513)		Mount Stromlo (No. 78259001)	
	*h*_2_	*l*_2_	*h*_2_	*l*_2_
LAGEOS-1	0.5764 ± 0.0007	0.0744 ± 0.0004	0.5616 ± 0.0009	0.0646 ± 0.0005
LAGEOS-2	0.5758 ± 0.0007	0.0748 ± 0.0004	0.5609 ± 0.0009	0.0650 ± 0.0005
LAGEOS-1+LAGEOS-2	0.5756 ± 0.0005	0.0751 ± 0.0002	0.5601 ± 0.0006	0.0637 ± 0.0003
STELLA	0.5741 ± 0.0022	0.0334 ± 0.0014	0.5622 ± 0.0026	0.0212 ± 0.0020
		(unacceptable value)		(unacceptable value)
STARLETTE	0.5750 ± 0.0019	0.1785 ± 0.0013	0.5604 ± 0.0022	0.0093 ± 0.0018
		(unacceptable value)		(unacceptable value)
STELLA+STARLETTE	0.5742 ± 0.0015	not estimated	0.5618 ± 0.0017	not estimated

**Table 2:** The *X*, *Y*, *Z* coordinates of the Yarragadee and Mount Stromlo SLR stations estimated in two calculation versions.

*X*, *Y*, *Z* (m) ITRF2014	*X*, *Y*, *Z* (m) Estimated Version 1 (Using the Nominal Global Values of *h*_2_, *l*_2_)	*X*, *Y*, *Z* (m) Estimated Version 2 (Using Local Values of *h*_2_, *l*_2_ Proposed in this Paper)	Version 1 Minus Version 2 (m)
**YARRAGADEE (no. 70900513)**			
−2389007.5340	−2389007.5204 ± 0.0022	−2389007.5171 ± 0.0022	−0.0033
5043329.4474	5043329.4418 ± 0.0019	5043329.4377 ± 0.0019	0.0041
−3078524.2232	−3078524.1935 ± 0.0017	−3078524.1883 ± 0.0017	−0.0052
**MOUNT STROMLO (no. 78259001)**			
−4467064.7778	−4467064.7519 ± 0.0021	−4467064.7481 ± 0.0019	−0.0038
2683034.8865	2683034.8632 ± 0.0017	2683034.8582 ± 0.0017	0.0050
−3667007.3186	−3667007.3331 ± 0.0016	−3667007.3386 ± 0.0016	0.0055
